# Serum BPI as a novel biomarker in asthma

**DOI:** 10.1186/s13223-020-00450-0

**Published:** 2020-06-18

**Authors:** Chen Xingyuan, Qiu Chen

**Affiliations:** Department of Respiratory and Critical Care Medicine, Shenzhen People’s Hospital (The Second Clinical Medical College, Jinan University), Shenzhen Institute of Respiratory Diseases, Shenzhen, China

**Keywords:** Asthma, Biomarker, BPI

## Abstract

**Background:**

Neutrophils, eosinophils and inflammatory cells contribute to asthmatic inflammation. The anti-bactericidal/permeability-increasing protein (BPI), produced by neutrophils, peripheral blood monocytes or epithelial cells, can neutralize lipopolysaccharide activity and enhance phagocytosis regulation function. This study aimed to assess the clinical significance of BPI in asthmatic patients.

**Methods:**

We recruited 18 controlled asthma, 39 uncontrolled asthma and 35 healthy controls individuals. Clinical characteristics (age, gender, allergy history, body mass index (BMI) and smoking history), clinical indicators [whole blood count, forced expiratory volume in one second as percentage of predicted volume (FEV1% predicted), IgE level, high sensitivity C-reactive protein (hs-CRP) and fractional expiratory nitric oxide (FeNO)] and serum BPI levels were measured to compare among each group. We then evaluated the correlation between BPI, clinical characteristics and clinical indicators. Finally, linear regression analysis was performed to exclude the influence of other factors and to find the independent influencing factors of BPI.

**Results:**

Our results showed that the serum BPI levels increased by twofold in the controlled asthma group (12.83 ± 6.04 ng/mL) and threefold in the uncontrolled asthma group (18.10 ± 13.48 ng/mL), compared to the healthy control group (6.00 ± 2.58 ng/mL) (p < 0.001). We further found that serum BPI levels were positively correlated with the hs-CRP (p = 0.002). There was no significant association among BPI, age, gender, BMI, allergy, blood eosinophils, blood neutrophils, IgE, FeNO or FEV1% predicted.

**Conclusion:**

BPI levels were increased in asthma and positively correlated with hs-CRP. BPI as a potential asthma biomarker that still needs further research.

## Background

Asthma is a chronic airway inflammatory disease with a complex and varied clinical presentation, including wheezing, chest tightness and dyspnea. The variation of asthma symptoms depends on the interaction of susceptibility genes and environmental factors [1]. Currently, diagnosis and treatment of asthma are often based on symptoms and lung function test results [2]. However, these may not be able to predict future exacerbation. In addition, many primary care facilities are not equipped with spirometry to perform the necessary tests. As a result, research on asthma is shifting from symptoms and lung function test to focusing on cell profiles, protein analysis, genetic and biomarkers. There is an ongoing search for noninvasive and reliable biomarkers, with the aim to identify the phenotype to assist the physicians [3].

The pathophysiology of asthma exacerbations involves the recruitment of immune cells to the lungs, including neutrophils, macrophages, eosinophils, and mast cells [4]. In addition, polarization, adhesion, chemotaxis, the release of phagocytic reactive oxygen species and degranulation of neutrophils play essential roles in asthma exacerbation. Neutrophils activate and release inflammatory mediators, such as IL-6, IL-13, interferon-gamma, GM-CSF, and TGF-beta, which can aggravate the immune response and promote acute exacerbation of asthma [5].

The anti-bactericidal/permeability-increasing protein (BPI) is a secretory protease produced by neutrophils, peripheral monocytes, and epithelial cells. BPI has an important anti-bacterial effect on gram-negative bacteria, neutralizes lipopolysaccharide activity, enhances phagocytosis regulation and possess an anti-fungal effect [6]. Antineutrophil cytoplasmic autoantibodies against neutrophil granule bactericidal/permeability-increasing protein (BPI-ANCA) has been found in many inflammatory diseases, such as COPD, and can reduce the detrimental effect of BPI on gram-negative bacteria. Positive BPI-ANCA is associated with the inflammatory status in COPD patients and can be used as a potential biomarker to assess the disease severity [7]. In addition, microarray analysis reported high RNA levels of BPI in the blood of asthmatic patients [8]. Based on these findings, we hypothesized that BPI is elevated in asthmatic patients, especially in the uncontrolled asthma group. To test our hypothesis, we conducted an observational study to measure BPI levels in asthmatic patients and healthy control individuals. Next, we evaluated the correlation among BPI levels, clinical characteristics and clinical indicators between the groups. Spearman correlation coefficient analysis was completed to reveal the impact of the independent variables on the results. Finally, in order to exclude the influence of other factors and to find the independent factors of BPI, we carried out a linear regression analysis.

## Materials and methods

### Classification of study subjects

We enrolled patients with asthma from the 1st of August 2019 to the 10th of October 2019 at the Department of Respiratory Medicine, Second Clinical College of Jinan University. The diagnosis of asthma was made accordingly to the criteria of the Global Initiative for Asthma (GINA) 2015 [9]. We used asthma control test (ACT) scores to classify the groups: controlled asthma group ACT scores ranged between 20 to 24, uncontrolled asthma group ACT scored below 20. Ethical approval was obtained from the Ethics Committee of the College of Science, Second Clinical College of Jinan University. Participants completed a questionnaire, which contains demographic information. Anthropometry measurements, which included height (cm), weight (kg), age, gender, smoking and history of other allergic conditions were determined. Body mass index (BMI) was calculated as weight (kg) divided by height (cm) in squared meters.

### Pulmonary function tests

Pulmonary function tests were performed using a SYSTEM 21^®^ device (MINATO MEDICAL SCIENCE CO., Osaka, Japan) according to the criteria of the American Thoracic Society (ATS)/European Respiratory Society and the Japanese Respiratory Society. Pulmonary function tests were performed, and FEV1% pred was included for our analysis.

### Measurement of fractional exhaled nitric oxide (FeNO) level

The FeNO levels were measured using a NIOX MINO^®^ device (Aerocrine AB, Solna, Sweden) according to the manufacturer’s instructions and the ATS guidelines.

### Blood sample collection and cell counts

Fasting blood samples were drawn, centrifuged and serum was placed in plain polystyrene tubes on the same day. Serum samples were sent to the laboratory for storage at − 80 °C. Peripheral blood cell counts were performed on each patient. Serum IgE, hs-CRP and BPI were measured using ELISA kits according to the manufacturer’s instructions (Cusabio Biotech, P.R. China).

### Statistical analysis

Shapiro–Wilk test was used to evaluate the normality of the data. The data was expressed in terms of mean (+ SD) and median (quartile range) of the parametric and nonparametric data, respectively. For the parameter data, student’s t-test and one-way ANOVA were used to compare two or more groups of data. For non-parametric data, the Mann–Whitney test and Kruskal–Wallis test were used to compare two or more groups of data. The classification of data was compared by Chi-square test or Fisher’s exact test. Dunnett test and Steel test were used to compare the parametric and non-parametric data. Linear regression analysis and Spearman correlation coefficients were used for correlations. We applied the Bonferroni correction to determine significance in the setting of multiple comparisons. The p-value was also corrected with LD-adjusted Bonferroni correction. Statistical analysis were performed using SPSS version 19.0.0 (IBM, New York, USA) and Sigma Pro version 11.0 (Systat Software Inc., Illinois, USA).

## Result

### The clinical characteristics of each group

There were no significant differences in age, gender, smoking rate, allergic history and BMI among the groups (Table 1). The uncontrolled asthma group showed significant differences in BPI (p < 0.001), blood eosinophils (p = 0.033) and blood neutrophils (p < 0.001) levels compared to the control group. In addition, the uncontrolled asthma group showed differences in BPI (p = 0.046), FEV1% predicted (p = 0.045) and blood neutrophils (p = 0.001) levels when compared to the controlled asthma group. The BPI difference between controlled asthma group and controlled group was statistically significant (p = 0.001).

**Table 1:** Patient’s characteristics and the level of clinical indicators

	Uncontrolled asthma (n = 39)	Controlled asthma (n = 18)	Controlled (n = 35)
Age	50.51 ± 12.13	42.17 ± 16.06	48.13 ± 8.08
Male sex (%)	35.90	44.44	34.29
BMI (kg/m^2^)	22.09 ± 3.07	22.56 ± 5.03	22.55 ± 3.00
Smoker (%)	38.46	33.33	25.71
Allergic history (%)	24.32	22.22	14.28
Blood			
Eosinophils counts	0.33 ± 0.41 * 10^9^/L*	0.28 ± 0.19 * 10^9^/L	0.15 ± 0.61 * 10^9^/L
Neutrophils counts	5.71 ± 2.86 * 10^9^/L*^#^	3.65 ± 1.08 * 10^9^/L	3.59 ± 1.09 * 10^9^/L
Total IgE (IU/mL)	367.90 ± 461.46	235.76 ± 197.31	–
FeNO (ppb)	46.67 ± 55.80	30.10 ± 21.41	–
Serum BPI (ng/mL)	18.10 ± 13.48*^,#^	12.83 ± 6.04*	6.00 ± 2.27
hs-CRP	8.59 ± 14.88	8.82 ± 15.64	–
FEV1% pred	63.84 ± 27.05^#^	72.78 ± 25.79	–

Data are presented as mean ± SD. Data in the controlled and uncontrolled asthma groups were collected before inhaled corticosteroid (ICS) therapy. *p < 0.05 Comparisons were made among the three groups

*IgE* immunoglobulin E, *BPI* bactericidal/permeability increasing protein, *FEV1% predicted* forced expiratory volume in one second as percentage of predicted volume

* p < 0.05 versus healthy control group

^#^p < 0.05 versus controlled asthma group

### Quantitative assessment of BPI levels

**Fig. 1:**
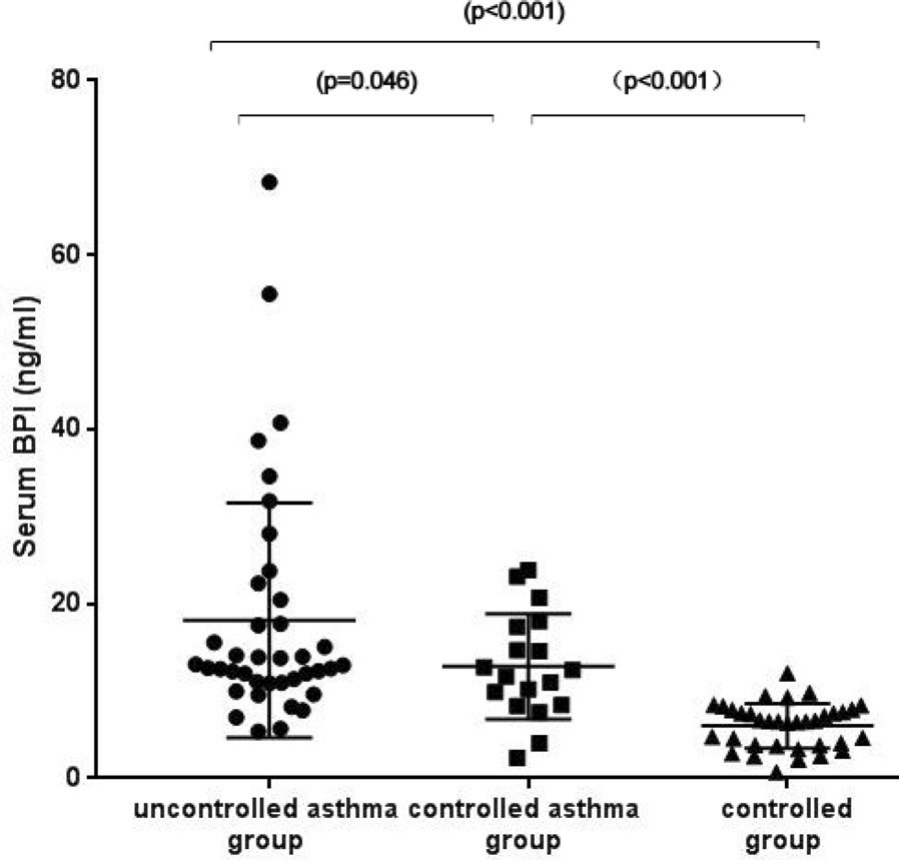
Comparison of BPI levels in control, controlled asthma and uncontrolled asthma groups. Values are presented as mean ± SD

The concentration of a molecule needs to represent a certain disease state in order for the molecule to be identify as a potential biomarker. We quantified the protein levels of BPI in each group by ELISA. We found a threefold induction of serum BPI level in the uncontrolled asthma group (18.10 ± 13.48 ng/mL, p = 0.046) and a twofold induction in the controlled asthma group (12.83 ± 6.04 ng/mL, p < 0.001) when compared to the healthy control group (6.00 ± 2.27 ng/mL, p < 0.001), as shown in (Fig. 1).

### Correlation between serum BPI and clinical indicators

**Fig. 2:**
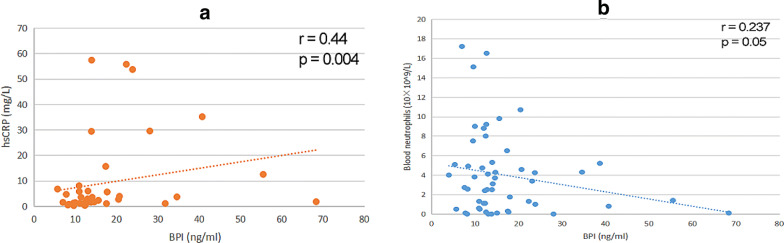
Correlation between serum BPI levels and biomarkers. **a** BPI and hs-CRP, **b** BPI and blood neutrophils

To investigate the clinical relevance of higher serum BPI levels, we checked the correlation of BPI levels with 10 clinical indicators in asthma patients. We measured the serum BPI by ELISA to compare with the clinical indicators. We adjust the p-value with LD-adjusted Bonferroni correction to reduce the increased risk of class I errors. The significance level was p < 0.005 after LD-adjusted bonferroni correction. We found that serum BPI accumulation was correlated with higher hs-CRP (p = 0.004) in asthma patients (Fig. 2a). After LD-adjusted Bonferroni correction, the correlation between BPI and hs-CRP was still confirmed in the asthma patients. The p-value of correlation between serum BPI and neutrophils was 0.05 (Fig. 2b). However, after applying the LD-adjusted Bonferroni correction, we could not confirm the significant correlation between BPI and blood neutrophils. No significant correlations were found among BPI and age, gender, BMI, allergy, blood eosinophils, IgE, FeNO, or FEV1% predicted (Table 2).

**Table 2:** Spearman’s correlation coefficients between BPI and other clinical indices

	Serum BPI (ng/mL)	
	rs	p-value
Age (years)	0.12	0.27
Gender	− 0.14	0.16
BMI (kg/m^2^)	− 0.11	0.93
Allergic history (%)	0.13	0.23
Blood eosinophils counts	− 0.004	0.98
Blood neutrophils counts	0.24	0.05
Total IgE (IU/mL)	0.03	0.89
hs-CRP	0.44	0.004*
FeNO (ppb)	− 0.07	0.70
FEV1% predicted	− 0.16	0.38

Spearman’s correlation coefficients between asthmatic and healthy individuals

*BMI* body mass index, *IgE* immunoglobulin E, *hs-CRP* high sensitivity C-reactive protein, *BPI* bactericidal/permeability increasing protein, *FeNO* fractional exhaled nitric oxide, *FEV1% predicted* forced expiratory volume in one second as percentage of predicted volume

* p < 0.05

In order to exclude any other factors influence and to find BPI independent factors, we performed linear regression analysis on BPI and hs-CRP according to Spearman’s correlation analysis. The p-value was corrected with LD-adjusted Bonferroni correction. The results indicated that hs-CRP was positively correlated with BPI after LD-adjusted Bonferroni correction (Table 3).

**Table 3:** Unary linear regression of BPI level in hs-CRP

Index	B	S.E.	Beta	t	p
Hs-CRP	0.16	0.05	0.42	3.0	0.005*

Linear regression analysis in BPI level and hs-CRP. High sensitivity C-reactive protein (hs-CRP)

* p < 0.005

## Discussion

We measured the BPI levels in 57 asthmatic patients (18 controlled asthma and 39 uncontrolled asthma) and 35 healthy individuals as controls to determine the role of serum BPI levels as a potential biomarker for the clinical management of asthma. Our data showed that serum BPI levels were the highest in the uncontrolled asthma group (18.10 ± 13.48 ng/mL) compared to the controlled asthma group (12.83 ± 6.04 ng/mL) and the healthy control group (6.00 ± 2.58 ng/mL) (p < 0.001). Further evidence showed that serum BPI levels were positively correlated with hs-CRP (p = 0.004). At the same time, unitary linear regression analysis indicated that hs-CRP was positively correlated with BPI. Therefore, BPI level maybe a new potential biomarker for asthma. The clinical application of BPI as an asthma biomarker needs to be further investigated.

In recent years, many asthma biomarkers have been reported to predict controlled asthma. High sputum eosinophils are associated with a better response to inhaled corticosteroids in asthmatic patients [10]. A biomarker of Th2 inflammation that can predict the response to IL-13 antibody, serum periostin is attracting increasing attention [11]. FeNO is also a biomarker of Th2 inflammation and is reported to be associated with clinical control of eosinophilic inflammation and asthma [12]. However, FeNO levels can be affected by a variety of factors, such as smoking and corticosteroids intake, so FeNO cannot predict or identify an acute exacerbation [13, 14]. New biomarkers are necessary because a large proportion of asthmatic patients exhibit significant airway inflammation. However, extracting biomarkers from sputum is difficult because people with stable asthma rarely produce sputum and many institutions have limited technical expertise to handle it [15].

Neutrophil activation generates smooth muscle contraction, membrane edema, increases vascular permeability, infiltration of white blood cells, and regulates immune response [16]. Activated neutrophils secrete a variety of cytokines, including IL-1, IL-6, TNF-alpha and PAF, which are associated with sustained airway injury, airway smooth muscle thickening and airway remodeling [17]. Previous studies reported BPI as a key marker of neutrophil activation [18, 19]; therefore, we hypothesized that the increase in BPI levels is associated with asthma.

Studies have shown that neutrophils, monocytes, eosinophils, epithelial cells and fibroblasts are involved in the inflammatory process of asthma [20]. Inflammatory cells can led to local tissue damage by releasing oxygen emission products, proteases and cationic substances, and can produce a variety of inflammatory mediators to affect the airway constriction of asthma. It has been reported that the expression of BPI on these cells is consistent with the role of BPI in the homing of cells to the inflammatory sites [21]. The role of BPI in apoptosis has also been previously confirmed [22]. Defects in apoptotic cell clearance during inflammation are associated with an inflammatory phenotype, such as a slow recovery of pulmonary inflammation [23]. These mechanisms may play a role in the increasing of airway inflammation in asthmatic patients.

In LPS-induced non-lethal sepsis mouse model, Zhou et al. demonstrated that BPI levels can significantly reduce the expression of TNF-alpha, MIP-2, and inhibit the effect of high-dose LPS intraperitoneal injection on cytokine response [24]. Balakrishnan et al. reported that BPI could neutralize the LPS-mediated activation of macrophage and block maturation of dendritic cells [25]. Furthermore, BPI prevented gram-negative bacteria to activate immune cells but did not affect the stimulating properties of gram-positive bacteria [26]. Another study suggested that BPI reduced mortality from endotoxin shock in mice [27].

Some literature supports the role of BPI, but the clinical significance of BPI is uncertain. One study found BPI is a significant minority target antigen for ANCAs in inflammatory bowel disease that seems related to colonic Crohn’s disease and disease activity in ulcerative colitis [28]. Another study identified elevated expression level of BPI in the liver cirrhosis patients than in healthy individuals, with a significant increase in patients who present a more severe disease [29]. In addition, outer basal epithelial cells of lacrimal gland ducts contain BPI, which occurs in a relatively high concentration in tears. BPI may have a substantial antibacterial role in human tears [30].

In this study, we demonstrated for the first time an elevated expression level of BPI in asthma patients. We initially found serum BPI levels of both the controlled and the uncontrolled asthma group were significantly higher compared to the healthy control group. However, we found tremendous overlap of the BPI level among the groups. A possible reason for this overlap could be insufficient sample size or the classification of asthma by ACT results was not significantly associated with eosinophilic or neutrophilic inflammation. In addition, a previous study demonstrated that eosinophilic or neutrophilic inflammation could persist in the controlled asthma patients despite the fact that the condition was controlled [31]. Previous studies have reported significant increases in serum hs-CRP expression in patients with severe persistent asthma [32]. Meanwhile, Razi et al. suggested that the serum hs-CRP level of patients with acute asthma was higher compared to healthy controls [33]. In our study, positive correlation between BPI and hs-CRP was found in the asthma patients after LD-adjusted Bonferroni correction. This suggests that the serum BPI level may predict controlled status of asthma. On the other hand, correlations between BPI and blood neutrophils did not meet the criteria for significance after LD-adjusted Bonferroni correction. To determine whether there is a difference in BPI between neutrophil asthma and eosinophil asthma, it will be necessary to increase the patient population. At the same time, patients with neutrophilic asthma and type 2 asthma need to be separated and compared between groups. Furthermore, there was no significant association among BPI, age, gender, BMI, allergy history, blood eosinophils, IgE, FeNO, or FEV1% pred. Our data showed that there was no correlation between hs-CRP and FEV1% predicted, consistent with Ramirez et al. data [34]. Our data suggested that BPI levels may not be related to age, gender, history of allergies, IgE, FeNO or BMI in asthma. FEV1% predicted was associated with the severity of bronchial asthma based on previous studies [35]. Therefore, BPI level may not be used as a clinical assessment of asthma severity. According to the Spearman correlation analysis results, we further carried out a linear regression analysis. These results suggest that BPI level was positively correlated with hs-CRP after LD-adjusted Bonferroni correction. Since previous studies have shown that hs-CRP indicates the presence of infection [36], we speculate that infection may associate with BPI protein in asthmatic patients. Taken together, we found that BPI can identify asthma control status.

Our study has several limitations. The sample size was relatively small to perform the necessary multi-factor analysis. A completely different longitudinal study is required to determine whether BPI can be used as a biomarker to predict exacerbation of asthma. In addition, we are unable to frequently measure BPI levels during the study period, which inhibits our ability to determine the effects of clinical intervention on BPI expression changes. Therefore, further research is needed to clarify the practical application value of BPI as a new biomarker in a clinical setting.

## Conclusions

In this study, we found that BPI can identify asthma control status. Serum BPI levels may be a biomarker that predicts asthma progression and can be used as a rapid and easily implemented clinical biomarker.

## Data Availability

All datasets used and/or analysed during the current study are available from the corresponding author on reasonable request.
